# Gas-phase amination of aromatic hydrocarbons by corona discharge-assisted nitrogen fixation

**DOI:** 10.1038/s41598-021-82190-8

**Published:** 2021-02-02

**Authors:** Shanshan Shen, Yunfeng Chai, Qirong Shen, You Jiang, Xiang Fang, Yuanjiang Pan

**Affiliations:** 1 Department of Chemistry, Zhejiang University, Hangzhou, 310027 People’s Republic of China; 2 National Institute of Metrology, Beijing, 100013 People’s Republic of China; 3 Hangzhou Wahaha Group Co. Ltd., Hangzhou, 310018 People’s Republic of China

**Keywords:** Mass spectrometry, Physical chemistry

## Abstract

This paper reports on the gas-phase amination reaction of aromatic hydrocarbons occurring under corona discharge conditions with N_2_ gas as the nitrogen source. The corona discharge device within an atmospheric pressure chemical ionization source was employed to achieve the plasma-assisted N_2_ fixation, and the coupled ion trap mass spectrometer (IT-MS) was used to detect positively charged product ions. In the model case, under APCI conditions, unusual product ions, [M + 16]^+^ and [M + 14]^+^, were observed. Based on the high resolution MS data and tandem mass spectrometric information, [M + 16]^+^ was confirmed to be protonated *p*-toluidine and [M + 14]^+^ was confirmed to be *p*-methylphenylnitrenium ion. According to the experimental results of the isotopic labelling and substituent effect, one feasible mechanism is proposed as follows. Firstly, N_2_ is activated by plasma caused via the corona discharge and then electrophilically attacks toluene, yielding the key intermediate, *p*-methylphenylnitrenium; secondly, the intermediate undergoes double-hydrogen transfer reaction to give rise to the final product ion, protonated *p*-toluidine. This study may provide a novel idea to explore new and green method for the synthesis of anilines.

## Introduction

The activation and fixation of atmospheric N_2_, a readily available but chemically inert substance, is critical to life and human activities^1^. There are mainly three pathways to convert atmospheric N_2_ gas to bioavailable nitrogen (NH_4_^+^ and NO_3_^-^), namely natural nitrogen fixation by means of lightning, biological nitrogen fixation with the assistance of the nitrogenase, and industrial nitrogen fixation known as Haber process^2^.

Artificial activation and fixation of N_2_ gas at mild conditions is a challenging scientific endeavor. Intensive efforts have been put forward to design and synthesize specific biomimetic catalysts of nitrogenase^3–7^. Yandulov and Schrock^8^ used a molybdenum amido complex to catalyze the reduction of N_2_ to NH_4_^+^. Misawa et al.^9^ developed a plasmon-induced technique for ammonia synthesis from N_2_ that responded to visible light through a strontium titanate (SrTiO_3_) photoelectrode loaded with gold nanoparticles. Masashi Hattori^10^ et al. reported a stable electron-donating heterogeneous catalyst, cubic CaFH to produce ammonia from N_2_ and H_2_ gases at 50 °C with an energy of 20 kJ·mol^−1^.

In addition to the aforementioned condensed-phase reactions, intriguing activation methods performed in the gas phase by applying high-energy source, e.g. electric field or plasma, have been preliminarily explored as well. For instance, Cooks’s group^11^ reported the direct insertion of nitrogen atom from N_2_ gas into C–C bonds of saturated alkanes to form iminium cations in a strong electric field, in which a paper-spray ionization source and a heated nitrogen atmosphere were employed. This method was further applied to a variety of hydrocarbons including branched alkanes as well as functionalized alkanes^12^. Similar reactions were also observed by Hiraoka’s group^13^. Zhang’s group^14^ observed that, in low-temperature plasma, one carbon atom in benzene could be directly replaced with nitrogen atom producing pyridine through gas phase ion/molecule reaction. The underlying reaction pathway was proposed to involve a carbon replacement with N-containing species and the ring-opening and closing reactions. Schwarz’s group^15^ reported that bare Ta_2_^+^ in the highly diluted gas phase was able to mediate the formation of ammonia in a Haber–Bosch-like process starting from N_2_ and H_2_ at ambient temperature, and further experimental and mechanistic aspects were explored by FTICR-MS and quantum chemical calculations^16^.

This paper reported on the gas-phase amination reaction of aromatic hydrocarbons occurring under plasma conditions typically found in the sources of atmospheric pressure chemical ionization (APCI) mass spectrometers. The reaction is observed through examination of positively charged ions, which is suggestive of the formation of a cationic intermediate, protonated nitrenium. In the model reaction, N_2_ gas is activated under corona discharge conditions, and then aminates toluene to give rise to *p*-toluidine, via the key intermediate *p*-methylphenylnitrenium.

## Results and discussion

**Figure 1:**
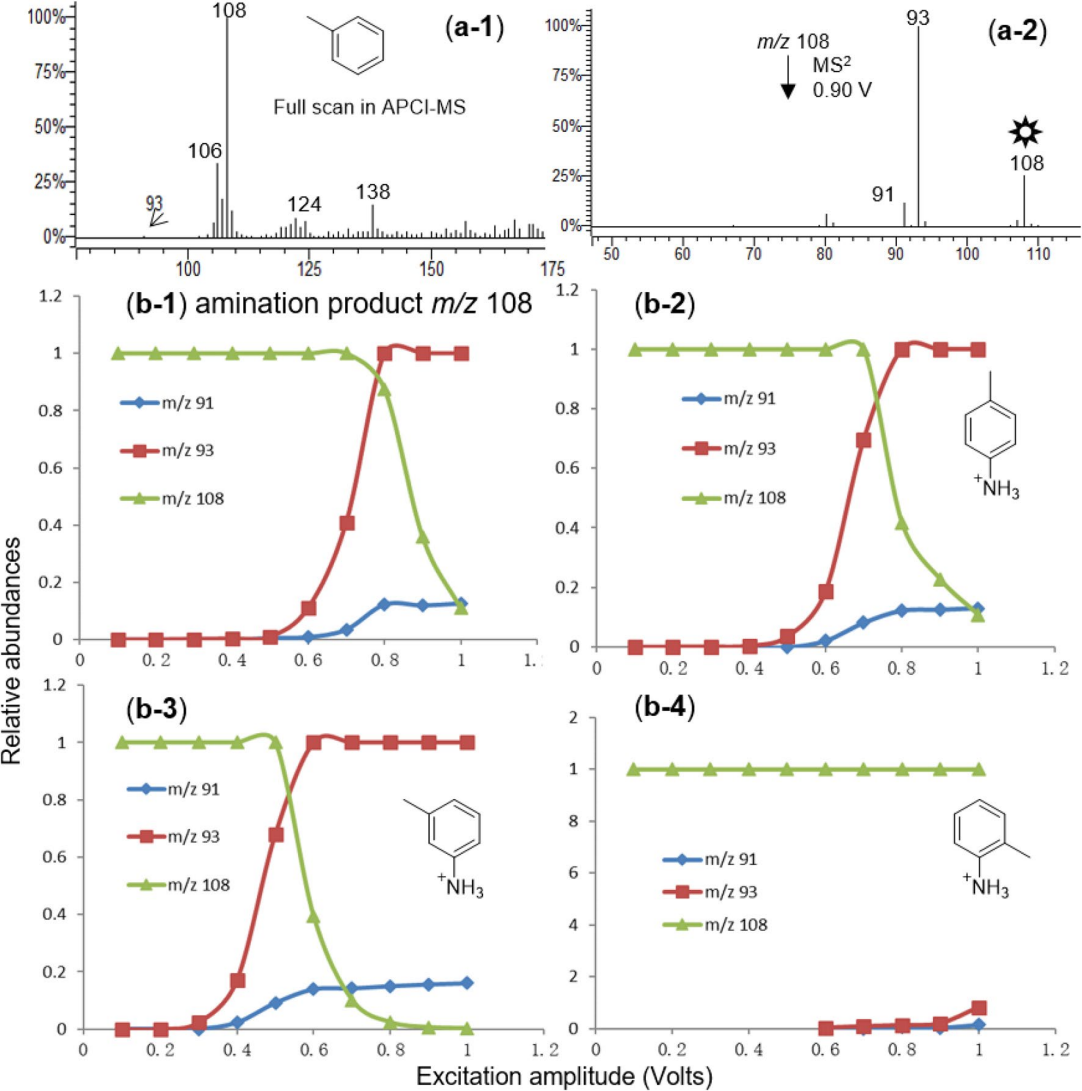
Full-scan and tandem mass spectra of the [M + 16]^+^ ion of toluene (**a-1**, **a-2**) under APCI conditions; and breakdown curves generated by abundances of selected fragment ions *versus* excitation amplitudes of the [M + 16]^+^ ion of toluene (**b-1**) and three positional protonated toluidine (**b-2**, **b-3** and **b-4**).

The corona discharge was achieved by the commercially available atmospheric pressure chemical ionization (APCI) source coupled with an ion trap mass spectrometer (IT-MS). The experiment was carried out by introducing toluene/CH_3_OH (1:1) solution into the APCI source and the reaction products were on-line monitored by the IT-MS detector in the positive ion mode. The results were recorded as shown in Fig. 1(a-1). The conventional quasi-molecular ion peaks of toluene (Mw = 92), the radical cation (M^+·^, *m/z* 92) and the protonated molecule ([M + H]^+^, *m/z* 93), have relatively low abundances. By contrast, the principal feature is the base peak at *m/z* 108 corresponding to [M + 16]^+^, which is not the conventional quasi-molecular ion peak generated under APCI conditions. Moreover, unusual ion peaks at *m/z* 106 ([M + 14]^+^) and *m/z* 138 ([M + 46]^+^) were observed as well. The abundances of these unconventional ions are relatively intense but they are unidentifiable in the case of electrospray ionization MS. We speculated that they were derived from the in-source reaction of toluene under APCI conditions. Our further work thus focused on these unusual ions.

### Chemical structure determination

Previous investigation by Cooks’s group^17^ of the behavior of benzene under APCI conditions employed air as matrix gas and produced ionized phenol ([M + 16]^+^) and protonated phenol ([M + 17]^+^). Isotopic labelling experiments indicated that O_2_ gas from the air was incorporated in the hydroxylation reaction. Thus, at first, we presumed that the unusual ion [M + 16]^+^ observed in our study was generated from the hydroxylation of toluene to form the [M + O]^+·^ radical cation. Nevertheless, high-resolution mass spectrometric data (Table 1) indicate that compared with toluene, the elemental composition of *m/z* 108 is [M + NH_2_]^+^, which was speculated to be the amination product. Meanwhile, the elemental compositions of *m/z* 106 and *m/z* 138 were calculated as [M + N]^+^ and [M + N + CH_3_OH]^+^, respectively. In view of methanol as the solvent employed, it was presumed that *m/z* 138 was derived from the association of *m/z* 106 and a methanol molecule.

**Table 1:** High resolution APCI-( +)MS data of the unusual ions derived from toluene/methanol.

Nominal *m/z*	Experimental mass	Theoretical mass	Relative error (ppm)	Elemental composition
106	106.0654	106.0657	− 2.8	C_7_H_8_N ([M + N]^+^)
108	108.0810	108.0808	− 2.8	C_7_H_10_N ([M + NH_2_]^+^)
138	138.0913	138.0919	− 4.3	C_8_H_12_ON ([M + N + CH_3_OH]^+^)

**Figure 2:**
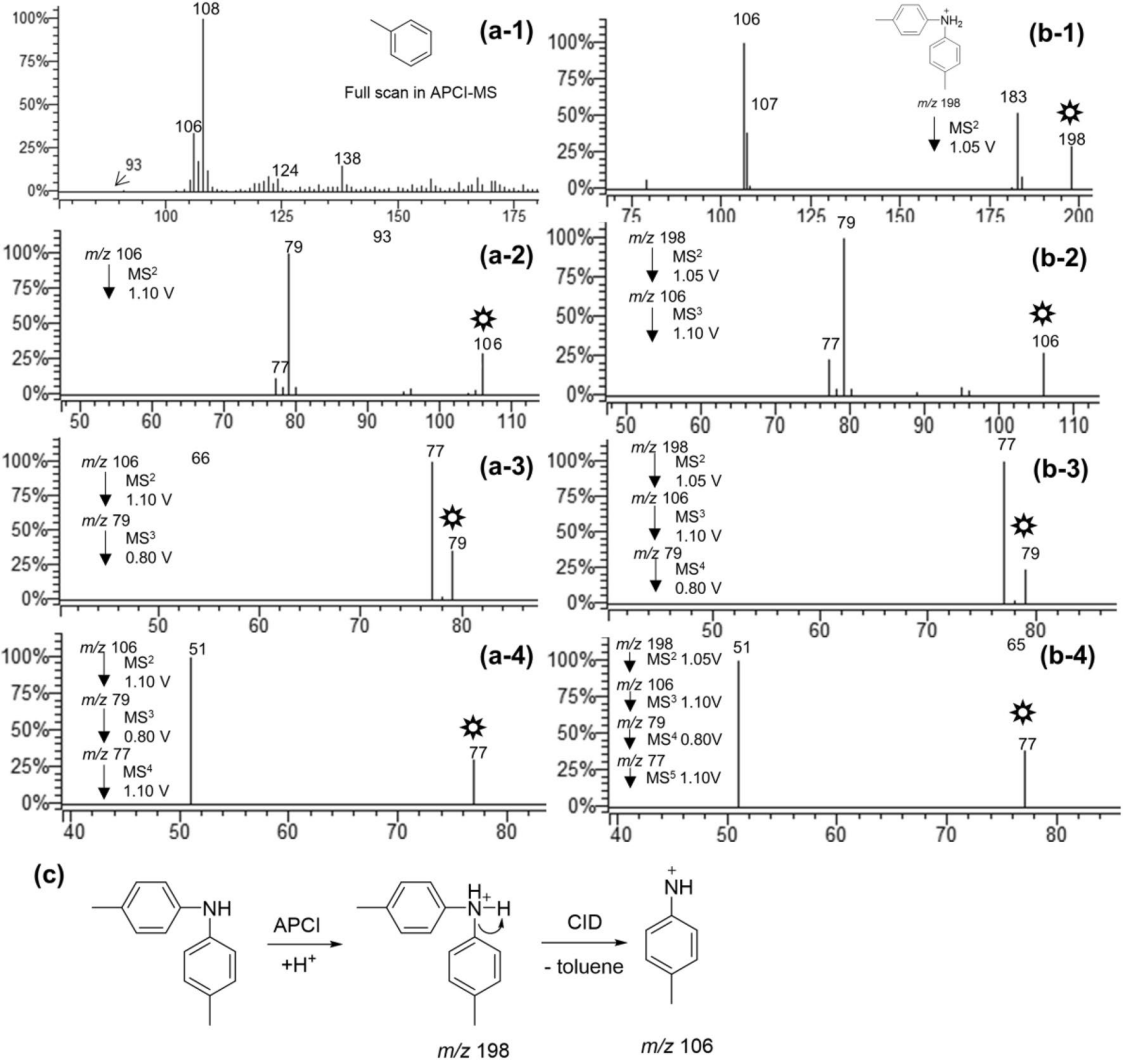
Full-scan and multistage mass spectra of *m/z* 106 from toluene (**a**), multistage mass spectra of protonated di-*p*-tolylamine (**b**), the generative method for the authentic *p*-methylphenylnitrenium (**c**).

**Figure 3:**
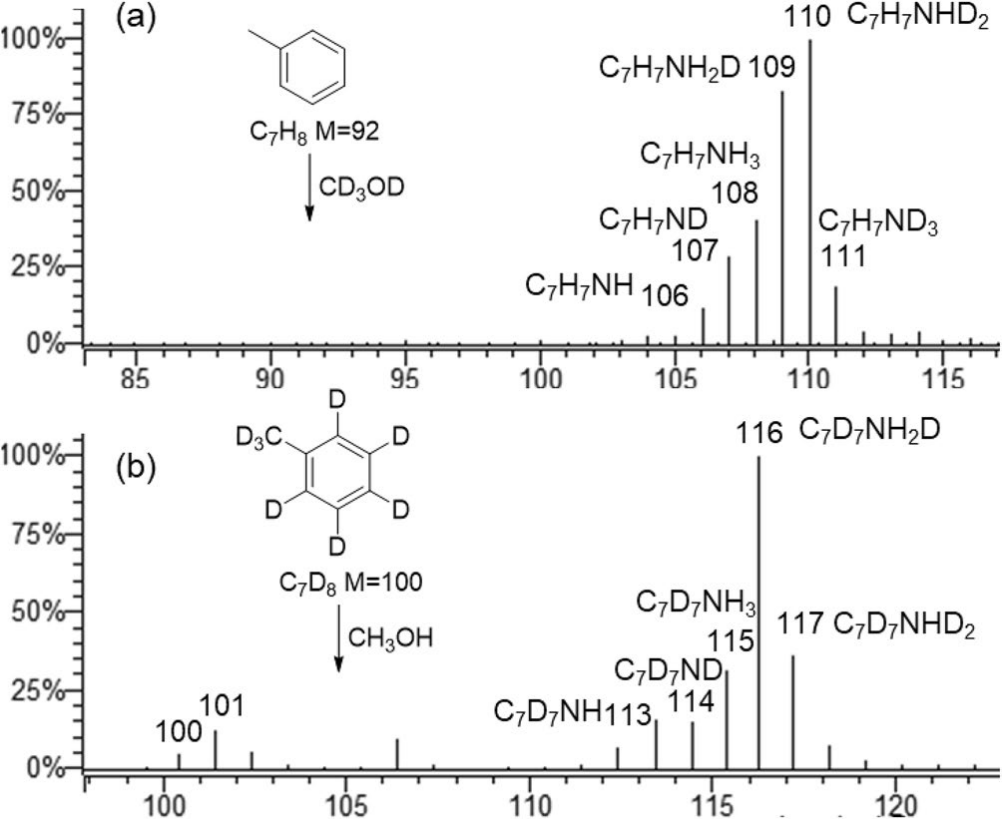
Full-scan APCI mass spectra of toluene/CD_3_OD (**a**) and *d*_8_-toluene/CH_3_OH (**b**).

**Figure 4:**
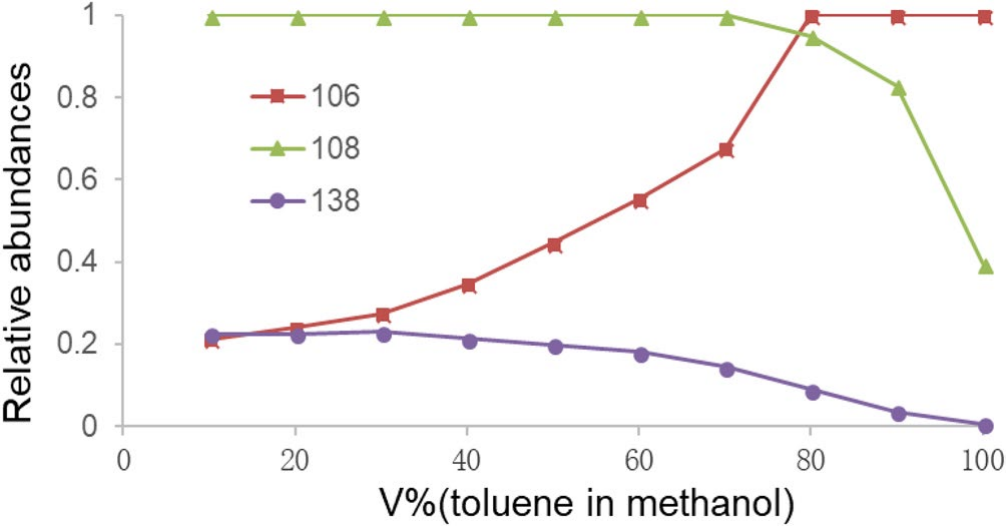
Relative abundances of the product ions along with the toluene proportion.

In order to further confirm the chemical structures of these unusual ions, tandem mass spectra were recorded and investigated. In the fragmentation of *m/z* 108 (Fig. 1(a-2)), two product ions resulting from the loss of methyl radical (*m/z* 93) or ammonia (*m/z* 91) were observed. The relative abundance of product ion *m/z* 91 is much lower than that of *m/z* 93, indicating that loss of ammonia is much more difficult than methyl radical, which means that the amino group is most probably attached to the phenyl ring, likely protonated toluidine. Within toluidine, the conjugation effect between the lone pair electrons of the nitrogen atom and the phenyl ring strengthens the C-N bond energy of the aromatic amino group, which makes the loss of ammonia in the fragmentation of protonated toluidine more difficult. Moreover, there are three positional isomers of toluidine, i.e. *ortho*-, *meta*- and *para*-toluidine. To further determine the chemical structure of *m/z* 108, we turned to the breakdown curve. Commonly, by evaluating the variation tendency of the characteristic fragment ions over collisional energies, the breakdown curve is widely applied to demonstrating the stereochemistry and identifying isomers and tautomers^18–20^. In this study, three characteristic ions (*m/z* 91, 93, 108) in the fragmentation of the unusual *m/z* 108 were selected to plot the breakdown curve as depicted in Fig. 1(b-1), which were further compared with that of three authentic protonated toluidine isomers, i.e. *para*- (Fig. 1(b-2)), *meta*- (Fig. 1(b-3)), and *ortho-* toluidine (Fig. 1(b-4)). As presented, the curve pattern of the unusual *m/z* 108 (Fig. 1(b-1)) closely resembles that in Fig. 1(b-2), which confirms that the chemical structure of the unusual *m/z* 108 is protonated *para-*toluidine. The aforementioned hydroxylation reaction is not applicable in this case, which was further evidenced by tandem mass spectrometrical analysis of the authentic methylphenol isomers (Fig. S2). It is probably due to the low O_2_ level during the APCI process.

We next tried to identify the chemical structure of *m/z* 106. Based on the chemical structure of *m/z* 108, *m/z* 106 was presumably attributed to *p*-methylphenylnitrenium ion. To test this hypothesis, multi-stage mass spectrometry was employed to compare the fragmentation pattern of *m/z* 106 with that of the authentic *p*-methylphenylnitrenium ion. The generation of the authentic *p*-methylphenylnitrenium is presented in Fig. 2c, which involves a neutral loss of toluene from protonated di-*p*-tolylamine in tandem mass spectrometry. As shown in Fig. 2, spectra on the left are the full scan and multi-stage mass spectra of *m/z* 106 derived from toluene (Fig. 2a), and the right are the multi-stage mass spectra of protonated di-*p*-tolylamine (Fig. 2b). Similar comparisons of multi-stage fragmentation information were made for the corresponding fragment ions, which confirms the chemical structure of *m/z* 106 to be *p*-methylphenylnitrenium.

To distinguish between a genuine toluene product ion and the trace contamination by *p*-toluidine, HPLC analysis of toluene and *p*-toluidine was performed (Fig. S3). No obvious impurities were found in the chromatogram of toluene. The results undoubtedly proved that the amination product ion was derived from toluene as a result of in-source reaction.

### Reaction mechanism exploration

In an effort of identifying the source of the hydrogen atoms involved in the amination reaction, APCI-( +)MS behaviors of deuterium-labeled substrates, including toluene/CD_3_OD and *d*_8_-toluene/CH_3_OH, were explored and MS data were recorded. Most remarkably, combined with the HR-MS data (Fig. S4), the spectrum of toluene/CD_3_OD (Fig. 3a) shows a base peak at *m/z* 110 which could be assigned to C_7_H_7_NHD_2_^+^ (relative error =  − 2.7 ppm). The inter-complementary experiment using *d*_8_-toluene/CH_3_OH (Fig. 3b) shows another base peak at *m/z* 116, corresponding to C_7_D_7_NH_2_D^+^ (relative error =  − 2.6 ppm). The elemental compositions of these two intense peaks indicate that, for the formation of aminium group of protonated *p*-toluidine, most probably one endogenous hydrogen atom from the benzen ring of toluene and two exogenous hydrogens from methanol serve as the hydrogen source. Meanwhile, the observation of ions at *m/z* 109 (C_7_H_7_NH_2_D^+^), 108 (C_7_H_7_NH_3_^+^) and 111 (C_7_H_7_ND_3_^+^) in Fig. 3a, as well as *m/z* 115 (C_7_D_7_NH_3_^+^) and 117 (C_7_D_7_NHD_2_^+^) in Fig. 3b, could be attributed as the result of H/D exchange with ambient labile protons (e.g. solvent or water in the air).

Next, we explored the effects of the relative proportions of the two substrates (toluene and methanol) on the amination reaction. The APCI mass spectra of toluene ranging from 10% to 100% were recorded and the relative abundances of the three unusual ions (*m/z* 106, 108 and 138) as a function of the toluene proportion were plotted. As shown in Fig. 4, in the case of pure toluene, the based peak was assigned to *p*-methylphenylnitrenium (*m/z* 106), the protonated *p*-toluidine (*m/z* 108) was identifiable, and the methanol associative ion (*m/z* 138) was barely visible. Along with the increase of the methanol proportion, the relative abundance of *m/z* 106 decreased, and the relative abundances of *m/z* 108 and 138 increased gradually. Based on the aforementioned results, it can be concluded that: (1) methanol plays a key role in the generation of *m/z* 108 and 138, but has little impact on *m/z* 106; (2) the formation of *m/z* 106 is prior to *m/z* 108 and 138, and further reaction between *m/z* 106 and methanol gives rise to the latter two ions; (3) *m/z* 108 is identifiable in the case of pure toluene, indicating that the solvent (e.g. methanol) is not the exclusive hydrogen source in the amination reaction.

With regard to the nitrogen source engaged in the amination reaction, we speculated that N_2_ gas was most likely involved in the formation of these unusual ions, since large amounts of N_2_ gas is employed for nebulization and drying of the solution when an APCI source operates. On the other hand, it is well-documented that, with the assistance of the plasma generated by corona dishcarge in APCI source, N_2_^+^ is regarded as the main initial charge carrier in discharges operating with N_2_ as matrix gas^21,22^ which may participate in subsequent gas-phase reactions during the ionization process.

Additionally, for other aromatics, amination reactions under APCI conditions are correlated to the electron effect of the substituent on the phenyl ring. Specifically, as listed in Table 2, aromatics bearing electron donating groups (e.g. anisole, ethylbenzene) generated abundant amination product ions (No. 1–3, 6); by contrast, those bearing electron-withdrawing groups (e.g. nitrobenzene, chlorobenzene) hardly underwent the amination reaction (No. 4, 5). These MS data suggest that the formation of the unusual product ions may be ascribed to the electrophilic reaction because the reactivity and orientation are highly consistent. It is noteworthy that, when the electron-donating effect of the substituent is strong enough (No. 7), the competitive protonation reaction during the ionization process will be very advantageous. In addition, probably because of the steric hindrance, the reactivity of the *ortho*- site is prohibited in this case.

**Table 2:** Amination results of different substituted benzene analogues.

No.	Aromatic hydrocarbon	[M + H]^+^	[M + N]^+^	[M + NH_2_]^+^
1	Toluene	93 (1)^a)^	106 (34)	108 (100)
2	Anisole	109 (69)	122 (27)	124 (100)
3	Ethylbenzene	107 (5)	120 (1)	122 (100)
4	^35^Chlorobenzene	113 (42)	126 (26)	128 (77)
5	Nitrobenzene	124 (100)	–^b)^	139 (1)
6	*p*-Xylene	107 (21)	120 (23)	122 (100)
7	1,3,5-Trimethoxybenzene	169 (100)	-	-

^a)^*m/z* (relative abundance %).

^b)^Relative abundance < 1%.

**Figure 5:**
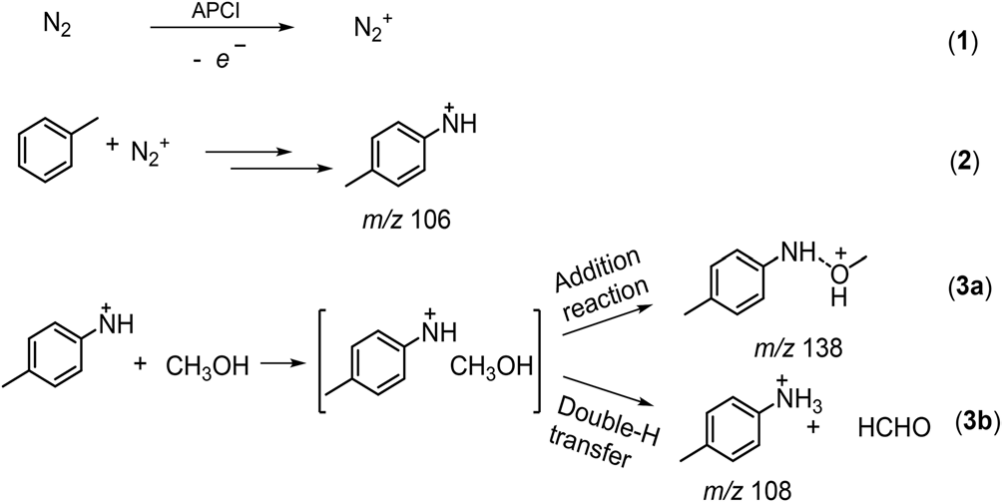
Proposed mechanism of the formation of protonated *p*-toluidine from toluene under the APCI condition.

Taking the aforementioned results into consideration, we propose a tentative reaction mechanism as shown in Fig. 5. Firstly, under APCI conditions, N_2_ gas is activated to produce reactive nitrogen species (e.g. N_2_^+^ in reaction 1), which subsequently electrophilically attacks the *para*-site of toluene to generated *p*-methylphenylnitrenium (2). The *p*-methylphenylnitrenium ion is a highly-reactive and ubiquitous intermediate that can react with nucleophile by addition reaction or hydrogen transfer^23,24^. Since solvent molecules are abundant in the APCI chamber, they are of great possibility to react with those reactive species inside the ionization source chamber. Taking the methanol as an example, it may solvate the *p*-methylphenylnitrenium ion to form an ion-neutral complex (INC) that may undergo (3a) addition reaction (*m/z* 138), or (3b) double-hydrogen transfer to generate protonated *p*-toluidine (*m/z* 108).

**Figure 6:**
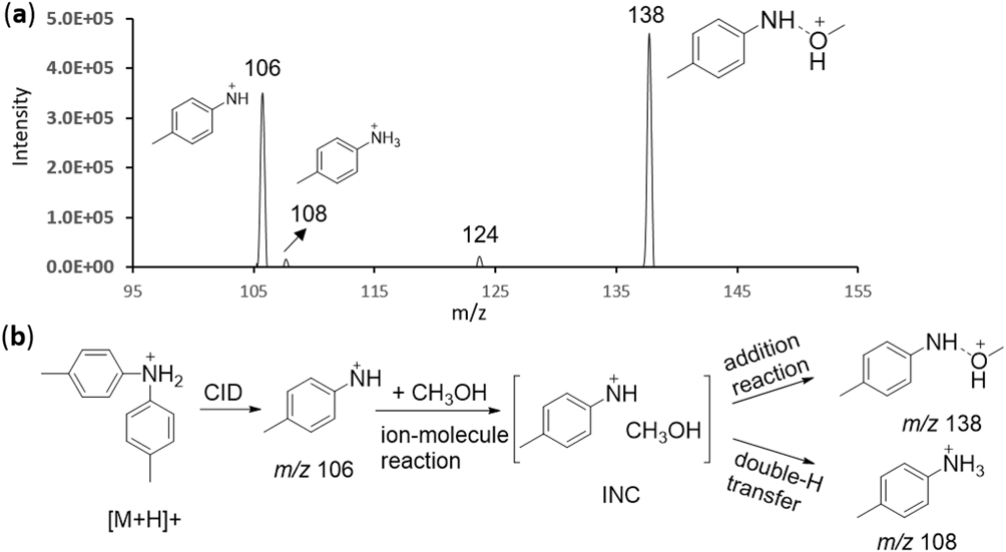
Gas phase ion/molecule reaction spectrum of *p*-methylphenylnitrenium ion and methanol, reaction time 1 s (**a**), and proposed reaction mechanism between *p*-methylphenylnitrenium ion (generated from the fragmentation of protonated di-*p*-tolylamine) and methanol (**b**).

At last, further experiments were performed comprehensively to support the proposed mechanism. On the one hand, the gas phase ion/molecule reaction between *p*-methylphenylnitrenium ion and methanol was performed in a home-made ion/molecule reactor^25^. The *p*-methylphenylnitrenium ion derived from the fragmentation of protonated di-*p*-tolylamine was isolated in the ion trap, which was followed by the introduction of methanol gas to examine its reactivity. The resultant mass spectrum is shown in Fig. 6a, and the reaction mechanism is proposed in Fig. 6b. The *p*-methylphenylnitrenium ion (*m/z* 106) reacted with MeOH to yield two identifiable product ions at *m/z* 108 (protonated *p*-toluidine, double-H transfer product) and *m/z* 138 (addition product). An ion/molecule complex is probably the intermediate in the formation of these two products, which plays a semblable role to the INC in reaction 3 (Fig. 5).

**Figure 7:**
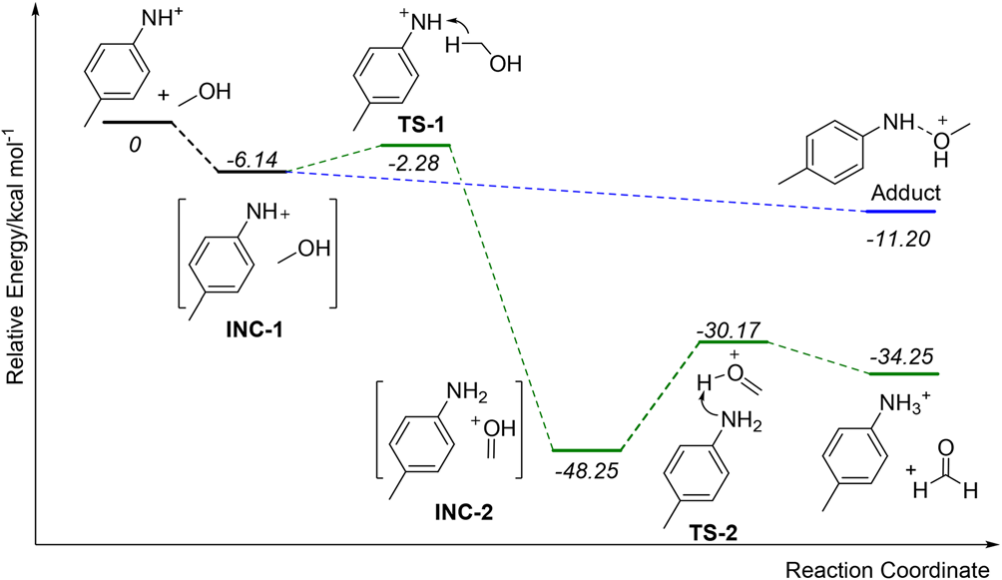
DFT potential energy diagram for the reaction mechanism of reaction **3**; the numbers in italics are relative energies in kcal mol^−1^.

Furthermore, a stepwise hydride transfer and proton transfer mechanism for the double-H transfer step was proposed, and density functional theory (DFT) calculations were applied to clarifying the proposed mechanism from the perspective of energetics. As depicted in Fig. 7, the *p*-methylphenylnitrenium ion interacts with methanol to form a complex INC-1 at first. It is followed by the hydride transfer via transition state TS-1 (only 3.86 kcal mol^−1^ higher than INC-1) to produce INC-2 (46.03 kcal mol^−1^ lower than TS-1). Subsequent proton transfer takes place via transition state TS-2 to generate protonated *p*-toluidine and formaldehyde. Compared with the starting materials, the final double-H transfer products yielded exergonically with an energy release of 34.25 kcal mol^−1^, which indicates that the proposed mechanism is thermodynamically accessible. Moreover, the addition reaction between *p*-methylphenylnitrenium and methanol is also favoured in terms of energy because the total energy of the adduct ion is 11.20 kcal mol^−1^ lower than the precursor ion. Although DFT calculations predict that the addition reaction is the thermodynamically disfavoured compared with the double-H transfer, it is still the most abundant product ion (Fig. 6a), probably due to the straightforward pathway of the formation of *m/z* 138.

## Conclusions

In this work, under selected APCI conditions, N_2_ gas can be activated by corona discharge, and undergo electrophilic substitution reactions of aromatic rings to trigger gas phase amination reactions of aromatics. One feasible mechanism was proposed: the formation of reactive intermediates, arylnitrenium ions, was speculated as the key step, followed by double-hydrogen transfer with ambient solvents to give rise to product ions. This study may provide a novel idea to explore new and green method for the synthesis of anilines.

## Materials and methods

### Materials

The reagents used were all commercial products and were used as received.

### Mass spectrometry and HPLC experiments

Mass spectrometry experiments were performed on a commercial ion trap mass spectrometer (Varian 500-MS) equipped with an APCI interface (Figure S7). Nitrogen was used as the nebulizing gas and drying gas. Toluene or substituted toluene was mixed with methanol (1: 1, v/v) and then was infused to the mass spectrometer with a syringe pump at a flow rate of 10 *μ*L min^−1^. The general instrumental conditions were described as follows. APCI in positive ion mode: spray chamber temperature 65 °C, nebulizer gas pressure 35 psi, drying gas pressure 10 psi, drying gas temperature 350 °C, vaporizer gas pressure 20 psi, vaporizer gas temperature 350 °C, corona current 5 *μ*A, spray shield voltage 600 V, capillary voltage 75 V, RF loading 75%, scan mass range 50–600 m*/z*. The multistage mass spectra were obtained with helium as the collision gas after isolation of the target precursor ions. The isolation window was 1 m*/z*. The fragmentation amplitude was adjusted properly to give the desired mass spectra.

The high-resolution mass spectrometry experiments were performed on a hybrid quadrupole-orbitrap mass spectrometer, Q-Exactive, combined with an UltiMate 3000 Binary Rapid Separation LC system (ThermoFischer Scientific, Bremen, Germany). A reversed-phase C18 column (hypersil DOLD, 100 × 2.1 mm, 3 μm) was used for chromatographic separation (column oven 35^◦^C). The isocratic mobile phase consisted of methanol–water (60:40, v/v) at a flow rate of 0.3 mL min^−1^. Toluene and *p*-methylaniline were dissolved in methanol to a final mix ratio of 5 ppm and 0.1 ppm, respectively. The injection volume is 3 *μ*L. Ionization by APCI interface in positive mode was performed using the following settings: discharge current 5 *μ*A, sheath gas (N_2_) flow rate 35 arb, auxiliary gas (N_2_) flow rate 10 arb, sweep gas (N_2_) flow rate 0 arb, heated capillary temperature 320 °C, vaporizer temperature 320 °C, S-lens radio frequency (RF) level 50.

The HPLC experiments were performed on Agilent 1260 Infinity II LC system. A reversed-phase C18 column (Agilent Poroshell 120 EC, 4.6 × 150 mm, 4 μm) was used for chromatographic separation. The optimized experimental conditions were: mobile phase 75:25 (v/v) CH_3_OH/H_2_O; flow rate 0.5 mL min^−1^; detection wavelength 254 nm; sample concentration 2 mg mL^−1^; injection volume, 2 μL.

### Ion/molecule reactions

Ion/molecule reaction experiment was performed on a home-made hybrid Q-Trap mass spectrometer based on two-dimensional (2-D) quadrupole ion trap mass analyzer, which was constructed at the National Institute of Metrology, which has been employed in our previous study^26^. The front quadruple was used for filtering the target ion (*p*-methylphenylnitrenium ion) into the back 2-D quadrupole ion trap. Isolation of the target ions was performed using the stored waveform inverse Fourier transform (SWIFT) method. Activation of the selected parent ion was accomplished with a single frequency, low amplitude (0.5–6 V) resonance excitation signal applied for 20–40 ms at a frequency corresponding to an activation q of 0.24–0.27. The product ion of interest, obtained by activation of the parent ion, was isolated to react with MeOH for a period of 1 s. The reagent MeOH was injected into the manifold, which was used to introduce the reagent into the helium buffer gas at a partial pressure of about 1.2 × 10^–5^ Torr.

### Theoretical calculation

Theoretical calculations were carried out using the Gaussian 03 package of programs^27^. Candidates structures of the reactants, products, intermediates and transition states were optimized by the RB3LYP methods at the basis set of 6–31 +  + G(d, p). All optimized structures were subjected to vibrational frequency analysis for zero-point energy (ZPE) correction to the temperature at 298.15 K and the pressure at 1.0 atm. The vibrational frequency analysis was carried out to ensure a transition state having only one imaginary vibrational frequency while a local or global minimum having no imaginary vibrational frequency. The minima connected by a given transition structure was confirmed by intrinsic reaction coordinate (IRC) calculations. The energies discussed here are the sum of electronic and thermal energies at kcal·mol^−1^.

## Supplementary Information

Supplementary Information.
