# Supplementary material for: Gas-phase amination of aromatic hydrocarbons by corona discharge-assisted nitrogen fixation
Source: Sci Rep. 2021 Feb 2;11:2841. doi: 10.1038/s41598-021-82190-8 (PMC7854734; doi:10.1038/s41598-021-82190-8)
Supplement: Supplementary file 1 — Supplementary Information. [file 41598_2021_82190_MOESM1_ESM.pdf]

Supplementary Information

Gas-phase amination of aromatic hydrocarbons by corona discharge-assisted nitrogen fixation

Shanshan Shen[1,3], Yunfeng Chai[1], Qirong Shen[1], You Jiang[2], Xiang Fang[2,*] & Yuanjiang Pan[1,*]

[1] Department of Chemistry, Zhejiang University, Hangzhou, 310027, P. R. China

[2] National Institute of Metrology, Beijing, 100013, P. R. China

[3] Hangzhou Wahaha Group Co. Ltd., Hangzhou, 310018, P. R. China

Address reprint requests to:

Yuanjiang Pan, panyuanjiang@zju.edu.cn;

Xiang Fang, fangxiang@nim.ac.cn

Figure Legends

Figure S1. High-resolution mass spectrum of toluene/methanol

Figure S2. Tandem mass spectra of the authentic methylphenol isomers

Figure S3. HPLC chromatograms of toluene (**a**) and toluene&*p*-toluidine mixture (**b**)

Figure S4. Full-scan high resolution APCI mass spectra of (**a**) toluene dissolved in CD_3OD, and (**b**) d_8-toluene dissolved in CH_3OH.

Figure S5. Figures, cartesian coordinates, total energies, zero point energy corrections of all optimized structures discussed in the text at the B3LYP/6-31++G (*d, p*) level

Figure S6. Relative abundances of the unusual product ions under different nebulizer gas pressure (**a**) and drying gas pressure (**b**)

Figure S7. Photos of APCI-IT-MS and corona discharge apparatus

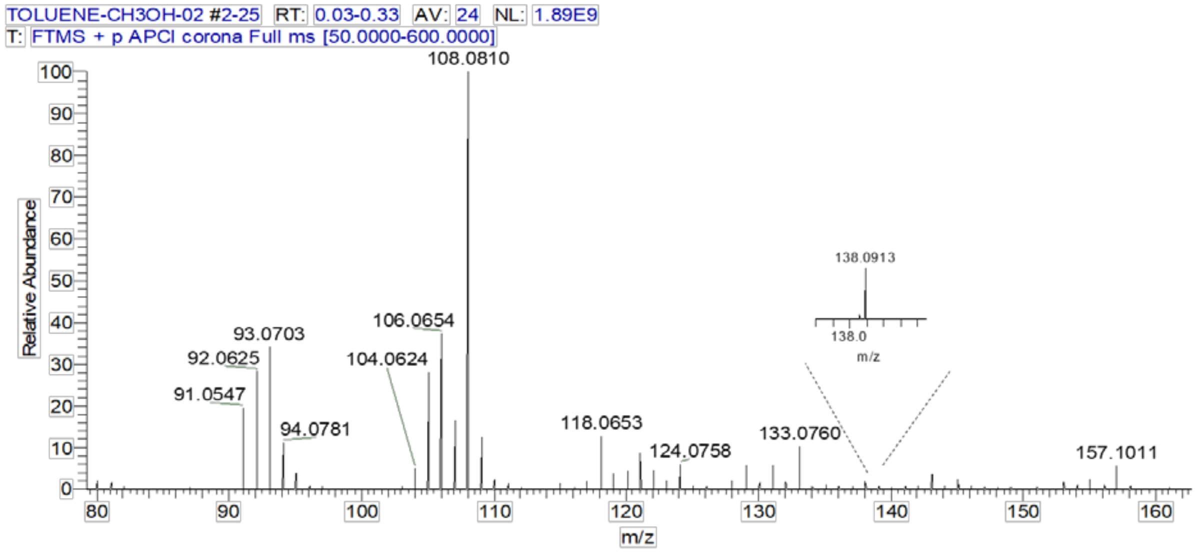

Figure S1. High-resolution mass spectrum of toluene/methanol

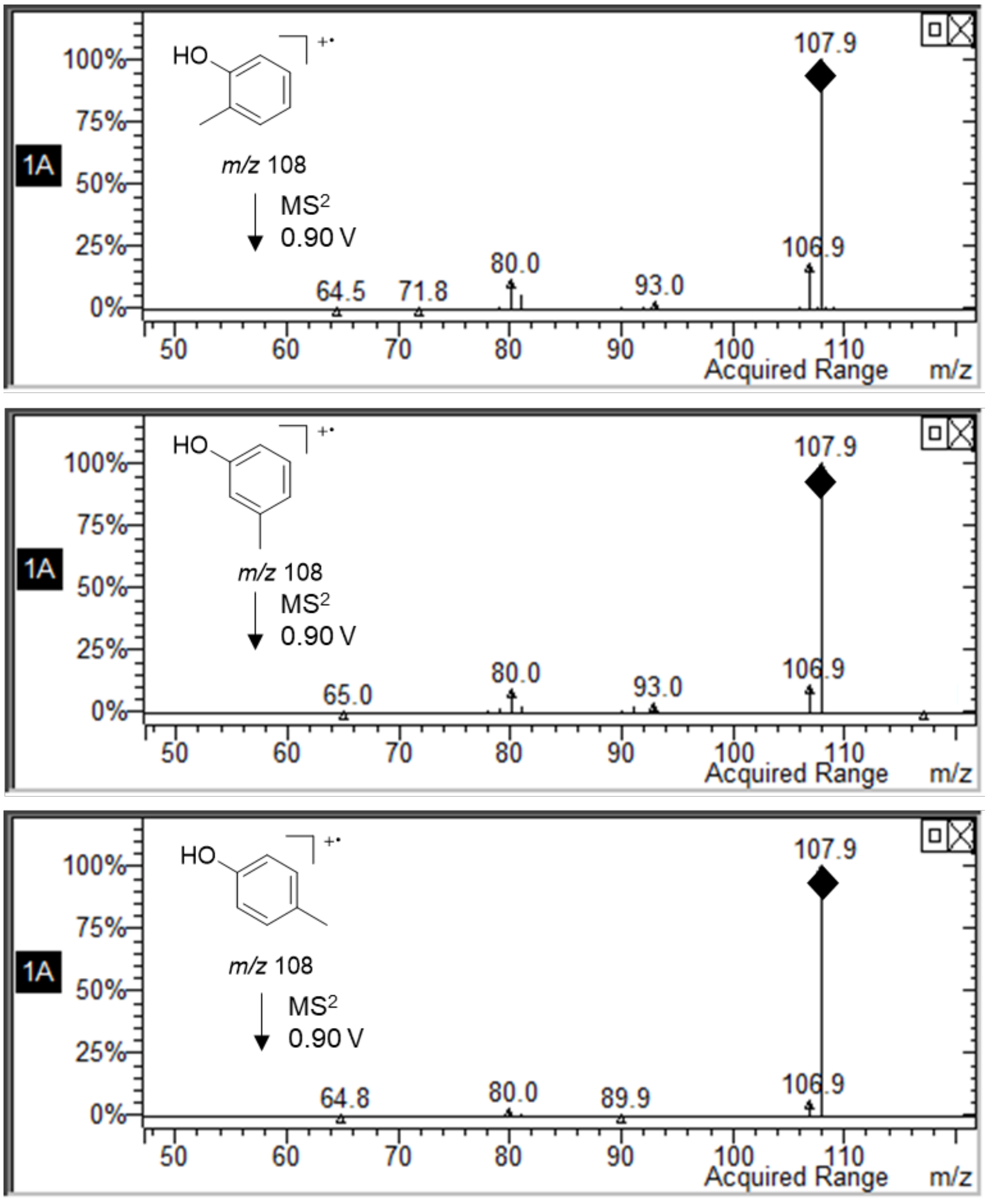

Figure S2. Tandem mass spectra of the authentic methylphenol isomers

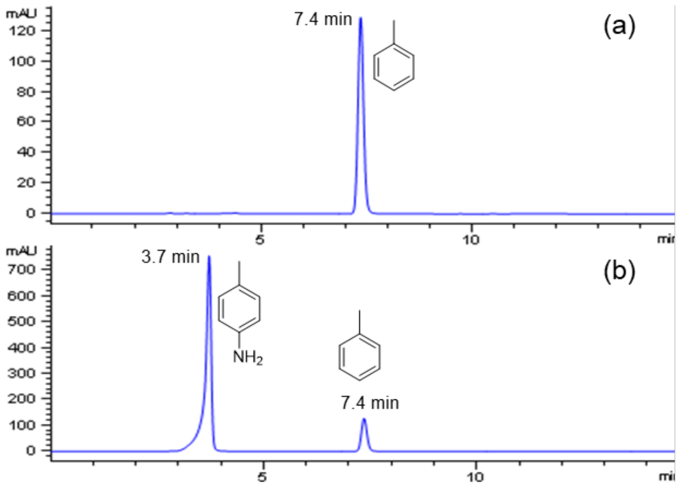

Figure S3. HPLC chromatograms of toluene (**a**) and toluene&*p*-toluidine mixture (**b**)

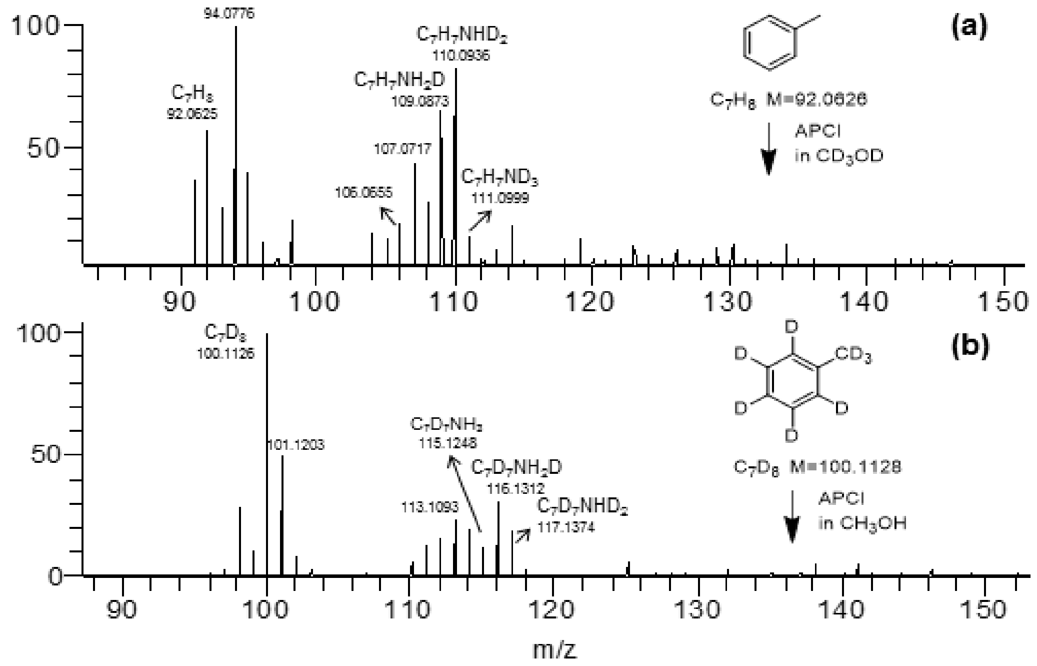

Figure S4. Full-scan high resolution APCI mass spectra of (**a**) toluene/CD_3OD and (**b**) d_8-toluene/CH_3OH.

Figure S5. Figures, cartesian coordinates, total energies, zero point energy corrections of all optimized structures discussed in the text at the B3LYP/6-31++G (*d*, *p*) level

MeOH

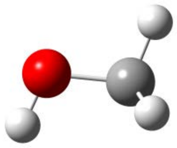

Center Number	Atomic Number	Atomic Type	Coordinates (Angstroms) X	Y	Z
1	6	0	0.668622	-0.020685	0.000000
2	1	0	1.083642	0.989600	-0.000002
3	1	0	1.029650	-0.546061	-0.894854
4	1	0	1.029650	-0.546058	0.894856
5	8	0	-0.749987	0.122537	0.000000
6	1	0	-1.154781	-0.753665	0.000000

Zero-point correction= 0.051213 (Hartree/Particle)

Thermal correction to Energy= 0.054546

Thermal correction to Enthalpy= 0.055491

Thermal correction to Gibbs Free Energy= 0.028451

Sum of electronic and zero-point Energies= -115.683757

Sum of electronic and thermal Energies= -115.680424

Sum of electronic and thermal Enthalpies= -115.679480

Sum of electronic and thermal Free Energies= -115.706519

No imaginary vibrational frequency

p-methylphenylnitrenium

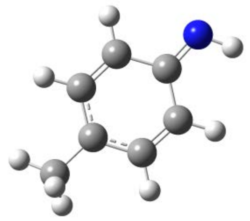

Center Number	Atomic Number	Atomic Type	Coordinates (Angstroms) X	Y	Z
1	6	0	-0.593637	-1.237727	-0.008423

2	6	0	0.767964	-1.252278	-0.001821
3	6	0	1.514546	0.010086	0.001318
4	6	0	0.756313	1.264134	-0.001234
5	6	0	-0.609812	1.247461	-0.008171
6	6	0	-1.314176	0.006157	-0.012542
7	1	0	-1.157043	-2.165698	-0.013181
8	1	0	1.323127	-2.186747	-0.000369
9	1	0	1.329938	2.186076	0.000712
10	1	0	-1.175413	2.173453	-0.013533
11	7	0	2.803254	0.112435	0.007996
12	1	0	3.270873	-0.803132	0.010049
13	6	0	-2.796844	-0.015190	0.003854
14	1	0	-3.193908	-0.807306	-0.640362
15	1	0	-3.129296	-0.266047	1.025688
16	1	0	-3.237179	0.946499	-0.262859

Zero-point correction= 0.132240 (Hartree/Particle)

Thermal correction to Energy= 0.139605

Thermal correction to Enthalpy= 0.140549

Thermal correction to Gibbs Free Energy= 0.100123

Sum of electronic and zero-point Energies= -325.891183

Sum of electronic and thermal Energies= -325.883818

Sum of electronic and thermal Enthalpies= -325.882874

Sum of electronic and thermal Free Energies= -325.923300

No imaginary vibrational frequency

Adduct

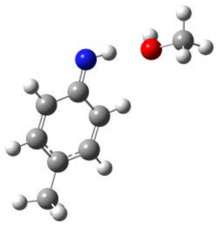

Center Number	Atomic Number	Atomic Type	Coordinates (Angstroms) X	Y	Z
1	6	0	-2.342560	0.984120	0.131365
2	6	0	-1.146899	1.643932	0.086744

3	6	0	0.102797	0.901119	-0.075822
4	6	0	0.035070	-0.559414	-0.185884
5	6	0	-1.174906	-1.189626	-0.137626
6	6	0	-2.384295	-0.438977	0.021166
7	1	0	-3.273548	1.529622	0.247682
8	1	0	-1.072533	2.724248	0.165108
9	1	0	0.969834	-1.101783	-0.307097
10	1	0	-1.239648	-2.270046	-0.221141
11	7	0	1.203400	1.581446	-0.111740
12	6	0	4.313239	-0.661568	0.568343
13	1	0	4.764506	-1.651273	0.452614
14	1	0	5.088981	0.109548	0.542223
15	8	0	3.318171	-0.430498	-0.454213
16	1	0	3.747972	-0.475645	-1.319363
17	1	0	2.029973	0.963064	-0.231801
18	1	0	3.794170	-0.615912	1.526851
19	6	0	-3.686596	-1.149555	0.103705
20	1	0	-3.877840	-1.394807	1.162127
21	1	0	-4.519504	-0.526881	-0.231490
22	1	0	-3.676643	-2.096463	-0.441774

Zero-point correction= 0.185686 (Hartree/Particle)

Thermal correction to Energy= 0.197837

Thermal correction to Enthalpy= 0.198781

Thermal correction to Gibbs Free Energy= 0.145328

Sum of electronic and zero-point Energies= -441.594237

Sum of electronic and thermal Energies= -441.582086

Sum of electronic and thermal Enthalpies= -441.581142

Sum of electronic and thermal Free Energies= -441.634595

No imaginary vibrational frequency

INC-1

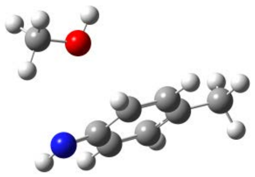

Center Number	Atomic Number	Atomic Type	Coordinates (Angstroms) X	Y	Z
1	6	0	-1.015870	-0.739927	0.986403
2	6	0	0.170674	-0.068760	1.144109
3	6	0	0.459194	1.127578	0.345612

4	6	0	-0.505756	1.515564	-0.687786
5	6	0	-1.659226	0.813223	-0.837390
6	6	0	-1.947461	-0.319901	0.006206
7	1	0	-1.247439	-1.601783	1.603162
8	1	0	0.902837	-0.348657	1.892609
9	1	0	-0.284287	2.379539	-1.308583
10	1	0	-2.388983	1.099503	-1.588585
11	7	0	1.533419	1.780845	0.641040
12	1	0	1.652139	2.613485	0.050746
13	6	0	-3.239491	-1.032354	-0.173135
14	1	0	-4.073750	-0.335957	-0.005719
15	1	0	-3.342244	-1.368381	-1.214265
16	1	0	-3.349928	-1.886731	0.494917
17	8	0	1.900437	-1.247252	-0.563245
18	1	0	1.773016	-2.161723	-0.849147
19	6	0	3.308916	-0.990437	-0.431254
20	1	0	3.786402	-1.706954	0.245032
21	1	0	3.803078	-1.013298	-1.408643
22	1	0	3.405854	0.013156	-0.009432

Zero-point correction= 0.185045 (Hartree/Particle)

Thermal correction to Energy= 0.197364

Thermal correction to Enthalpy= 0.198309

Thermal correction to Gibbs Free Energy= 0.144998

Sum of electronic and zero-point Energies= -441.586340

Sum of electronic and thermal Energies= -441.574020

Sum of electronic and thermal Enthalpies= -441.573076

Sum of electronic and thermal Free Energies= -441.626386

No imaginary vibrational frequency

TS-1

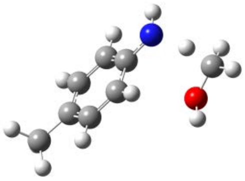

Center Number	Atomic Number	Atomic Type	Coordinates (Angstroms) X	Y	Z
1	6	0	-1.488675	0.979477	-0.738044
2	6	0	-0.202136	1.359750	-0.433869
3	6	0	0.539758	0.652860	0.569228
4	6	0	-0.064290	-0.484102	1.199725

5	6	0	-1.346624	-0.862145	0.867219
6	6	0	-2.088755	-0.138406	-0.099725
7	1	0	-2.055374	1.529871	-1.482549
8	1	0	0.256419	2.208419	-0.934793
9	1	0	0.497327	-1.006070	1.968885
10	1	0	-1.806415	-1.713892	1.358515
11	7	0	1.762545	1.030736	0.993565
12	1	0	2.039788	1.922607	0.566510
13	8	0	2.193921	-1.130589	-1.054399
14	6	0	3.221380	-0.447364	-0.480027
15	1	0	3.942127	-1.040152	0.091850
16	1	0	3.655866	0.268027	-1.178736
17	1	0	2.713102	0.260411	0.438507
18	1	0	2.047204	-1.989796	-0.630183
19	6	0	-3.496656	-0.524414	-0.423671
20	1	0	-4.187329	0.063140	0.198703
21	1	0	-3.745907	-0.307792	-1.465933
22	1	0	-3.690003	-1.579147	-0.215555

Zero-point correction= 0.182307 (Hartree/Particle)

Thermal correction to Energy= 0.192933

Thermal correction to Enthalpy= 0.193877

Thermal correction to Gibbs Free Energy= 0.144662

Sum of electronic and zero-point Energies= -441.578494

Sum of electronic and thermal Energies= -441.567867

Sum of electronic and thermal Enthalpies= -441.566923

Sum of electronic and thermal Free Energies= -441.616138

One imaginary vibrational frequency: -727.51

INC-2

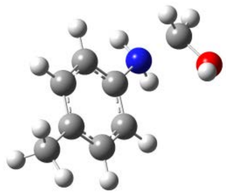

Center Number	Atomic Number	Atomic Type	Coordinates (Angstroms) X	Y	Z
1	6	0	1.380220	-1.129868	0.504459
2	6	0	0.025444	-0.910363	0.761999
3	6	0	-0.518736	0.331045	0.443032
4	6	0	0.248367	1.348734	-0.123061
5	6	0	1.598607	1.106934	-0.370135

6	6	0	2.188655	-0.131614	-0.061379
7	1	0	1.812801	-2.093718	0.754130
8	1	0	-0.581692	-1.695100	1.205092
9	1	0	-0.183172	2.317975	-0.361954
10	1	0	2.204072	1.896531	-0.804715
11	7	0	-1.962400	0.569787	0.674560
12	1	0	-2.293449	-0.038659	1.432314
13	8	0	-2.915360	-1.054919	-0.799860
14	6	0	-2.887543	0.291177	-0.551112
15	1	0	-2.493092	0.907265	-1.363123
16	1	0	-3.882893	0.621267	-0.245383
17	1	0	-2.231267	-1.321496	-1.431492
18	6	0	3.658197	-0.365280	-0.303159
19	1	0	4.252208	0.057238	0.516108
20	1	0	3.890323	-1.431125	-0.363977
21	1	0	3.991381	0.113603	-1.228098
22	1	0	-2.111643	1.535805	0.988583

Zero-point correction= 0.192103 (Hartree/Particle)

Thermal correction to Energy= 0.202258

Thermal correction to Enthalpy= 0.203202

Thermal correction to Gibbs Free Energy= 0.155363

Sum of electronic and zero-point Energies= -441.651293

Sum of electronic and thermal Energies= -441.641138

Sum of electronic and thermal Enthalpies= -441.640194

Sum of electronic and thermal Free Energies= -441.688034

No imaginary vibrational frequency

TS-2

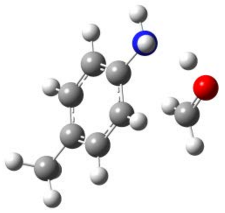

Center Number	Atomic Number	Atomic Type	Coordinates (Angstroms) X	Y	Z
1	6	0	-1.487224	1.063595	-0.597485
2	6	0	-0.190580	1.539485	-0.436759
3	6	0	0.711009	0.834968	0.372071
4	6	0	0.300079	-0.381144	0.973382
5	6	0	-1.034385	-0.818894	0.822502
6	6	0	-1.941119	-0.122229	0.028105

7	1	0	-2.177811	1.630180	-1.216278
8	1	0	0.114892	2.461311	-0.924230
9	1	0	0.947938	-0.880164	1.689780
10	1	0	-1.349125	-1.719009	1.342407
11	7	0	2.076598	1.221361	0.458683
12	1	0	2.261700	2.149860	0.084494
13	1	0	2.468506	1.155435	1.397174
14	6	0	1.579665	-1.457444	-0.798179
15	1	0	1.343522	-2.486504	-0.531967
16	1	0	0.957729	-0.924453	-1.515977
17	8	0	2.743725	-1.040541	-0.485762
18	1	0	2.750254	-0.000799	-0.360217
19	6	0	-3.366849	-0.580396	-0.147570
20	1	0	-4.064690	0.155757	0.266450
21	1	0	-3.615351	-0.701057	-1.207299
22	1	0	-3.547123	-1.533401	0.354575

Zero-point correction= 0.187000 (Hartree/Particle)

Thermal correction to Energy= 0.196812

Thermal correction to Enthalpy= 0.197757

Thermal correction to Gibbs Free Energy= 0.151878

Sum of electronic and zero-point Energies= -441.622133

Sum of electronic and thermal Energies= -441.612321

Sum of electronic and thermal Enthalpies= -441.611377

Sum of electronic and thermal Free Energies= -441.657255

One imaginary vibrational frequency: -260.41

p-toluidine

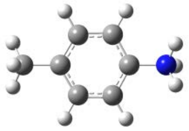

Center Number	Atomic Number	Atomic Type	Coordinates (Angstroms) X	Y	Z
1	6	0	1.222553	-1.206211	0.014844
2	6	0	-0.172434	-1.220264	-0.004168
3	6	0	-0.838709	0.000037	-0.014776
4	6	0	-0.172408	1.220018	-0.003597
5	6	0	1.222842	1.206207	0.015344
6	6	0	1.942862	0.000112	0.023105
7	1	0	1.757005	-2.151046	0.022309

8	1	0	-0.709674	-2.165345	-0.008657
9	1	0	-0.709580	2.165182	-0.007639
10	1	0	1.757131	2.151111	0.023203
11	7	0	-2.335782	-0.000006	0.001626
12	1	0	-2.710227	0.845437	-0.443479
13	1	0	-2.713156	-0.804946	-0.511234
14	1	0	-2.713861	-0.038198	0.956913
15	6	0	3.450389	-0.000264	0.011641
16	1	0	3.823713	-0.007018	-1.019681
17	1	0	3.854518	0.889232	0.501740
18	1	0	3.854054	-0.883741	0.512981

Zero-point correction= 0.158603 (Hartree/Particle)

Thermal correction to Energy= 0.166735

Thermal correction to Enthalpy= 0.167679

Thermal correction to Gibbs Free Energy= 0.124579

Sum of electronic and zero-point Energies= -327.144870

Sum of electronic and thermal Energies= -327.136738

Sum of electronic and thermal Enthalpies= -327.135793

Sum of electronic and thermal Free Energies= -327.178893

No imaginary vibrational frequency

Aldehyde

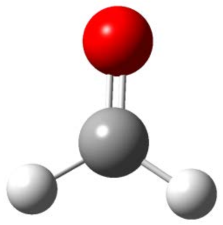

Center Number	Atomic Number	Atomic Type	Coordinates (Angstroms) X	Y	Z
1	6	0	0.000004	0.531628	0.000000
2	1	0	-0.941498	1.116945	0.000000
3	1	0	0.941439	1.117063	0.000000
4	8	0	0.000004	-0.677972	0.000000

Zero-point correction= 0.026670 (Hartree/Particle)

Thermal correction to Energy= 0.029538

Thermal correction to Enthalpy= 0.030482

Thermal correction to Gibbs Free Energy= 0.005001

Sum of electronic and zero-point Energies= -114.484959

Sum of electronic and thermal Energies= -114.482091

Sum of electronic and thermal Enthalpies= -114.481147

Sum of electronic and thermal Free Energies= -114.506628

No imaginary vibrational frequency

Toluene

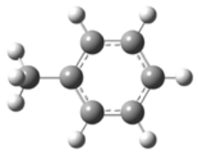

Center Number	Atomic Number	Atomic Type	Coordinates (Angstroms) X	Y	Z
1	6	0	-0.194919	1.203600	-0.009402
2	6	0	1.202467	1.206543	0.002060
3	6	0	1.907314	-0.000105	0.009077
4	6	0	1.202196	-1.206686	0.002061
5	6	0	-0.195106	-1.203499	-0.009403
6	6	0	-0.915699	0.000154	-0.011765
7	1	0	-0.733861	2.148173	-0.018661
8	1	0	1.739592	2.151053	0.001619
9	1	0	2.993461	-0.000227	0.014735
10	1	0	1.739178	-2.151278	0.001618
11	1	0	-0.734216	-2.147990	-0.018665
12	6	0	-2.427446	0.000068	0.009920

13	1	0	-2.834982	-0.880756	-0.496178
14	1	0	-2.807246	-0.009834	1.039547
15	1	0	-2.834775	0.890409	-0.479299

Zero-point correction= 0.127701 (Hartree/Particle)

Thermal correction to Energy= 0.133950

Thermal correction to Enthalpy= 0.134895

Thermal correction to Gibbs Free Energy= 0.096732

Sum of electronic and zero-point Energies= -271.461650

Sum of electronic and thermal Energies= -271.455401

Sum of electronic and thermal Enthalpies= -271.454457

Sum of electronic and thermal Free Energies= -271.492620

N_3^+

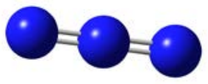

Center	Atomic	Atomic	Coordinates (Angstroms)		
Number	Number	Type	X	Y	Z
1	7	0	0.000000	0.000000	1.188344
2	7	0	0.000000	0.000000	-0.000001

3	7	0	0.000000	0.000000	-1.188344

Zero-point correction= 0.008542 (Hartree/Particle)

Thermal correction to Energy= 0.011520

Thermal correction to Enthalpy= 0.012464

Thermal correction to Gibbs Free Energy= -0.012887

Sum of electronic and zero-point Energies= -163.666875

Sum of electronic and thermal Energies= -163.663897

Sum of electronic and thermal Enthalpies= -163.662953

Sum of electronic and thermal Free Energies= -163.688304

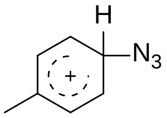

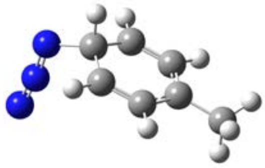

Center Number	Atomic Number	Atomic Type	Coordinates (Angstroms) X	Y	Z
1	6	0	1.247805	1.238944	-0.100674
2	6	0	-0.032015	1.256635	-0.572343
3	6	0	-0.032369	-1.256128	-0.573192
4	6	0	1.247494	-1.239124	-0.101524

5	6	0	1.915596	-0.000282	0.145123
6	1	0	1.765902	2.167287	0.119469
7	1	0	-0.560489	2.190926	-0.744774
8	1	0	-0.561111	-2.190159	-0.746223
9	1	0	1.765334	-2.167757	0.117958
10	6	0	3.291680	-0.000400	0.704386
11	1	0	3.213120	0.005301	1.804765
12	1	0	3.849293	-0.899431	0.431682
13	1	0	3.852203	0.894386	0.423408
14	6	0	-0.740508	0.000469	-0.941525
15	1	0	-0.663421	0.000799	-2.055225
16	7	0	-2.194869	0.000547	-0.708726
17	7	0	-2.527892	-0.000143	0.501283
18	7	0	-2.998230	-0.000694	1.534221

Zero-point correction= 0.140637 (Hartree/Particle)

Thermal correction to Energy= 0.149186

Thermal correction to Enthalpy= 0.150130

Thermal correction to Gibbs Free Energy= 0.106135

Sum of electronic and zero-point Energies= -435.326187

Sum of electronic and thermal Energies= -435.317638

Sum of electronic and thermal Enthalpies= -435.316694

Sum of electronic and thermal Free Energies= -435.360688

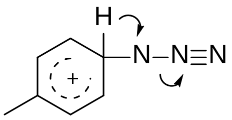

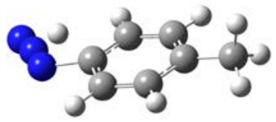

Center Number	Atomic Number	Atomic Type	Coordinates (Angstroms) X	Y	Z
1	6	0	-1.658363	1.163079	-0.070083
2	6	0	-0.307920	1.462618	-0.041050
3	6	0	0.193271	-0.961076	0.086927
4	6	0	-1.164373	-1.217250	0.045814
5	6	0	-2.115451	-0.171571	-0.031703
6	1	0	-2.376691	1.973208	-0.144631
7	1	0	0.053838	2.484581	-0.093279
8	1	0	0.907688	-1.777264	0.139576
9	1	0	-1.506206	-2.247437	0.064709
10	6	0	-3.582408	-0.480690	-0.051444
11	1	0	-3.787435	-1.424064	-0.564268
12	1	0	-3.953617	-0.583735	0.977378
13	1	0	-4.156998	0.316688	-0.528141
14	6	0	0.617436	0.393811	0.085999
15	1	0	1.307358	0.692857	1.125319

16	7	0	2.076842	0.833856	0.061676
17	7	0	2.930603	-0.098717	-0.052432
18	7	0	3.795257	-0.816333	-0.169734

Zero-point correction= 0.136462 (Hartree/Particle)

Thermal correction to Energy= 0.145666

Thermal correction to Enthalpy= 0.146610

Thermal correction to Gibbs Free Energy= 0.101392

Sum of electronic and zero-point Energies= -435.287061

Sum of electronic and thermal Energies= -435.277858

Sum of electronic and thermal Enthalpies= -435.276914

Sum of electronic and thermal Free Energies= -435.322132

N_2

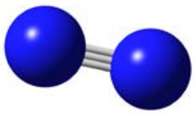

Center Number	Atomic Number	Atomic Type	Coordinates (Angstroms) X	Y	Z
1	7	0	-7.912910	0.210643	0.412290
2	7	0	-9.018035	0.210643	0.412290

Zero-point correction= 0.005592 (Hartree/Particle)

Thermal correction to Energy= 0.007953

Thermal correction to Enthalpy= 0.008897

Thermal correction to Gibbs Free Energy= -0.012857

Sum of electronic and zero-point Energies= -109.524187

Sum of electronic and thermal Energies= -109.521827

Sum of electronic and thermal Enthalpies= -109.520883

Sum of electronic and thermal Free Energies= -109.542636

Toluene radical cation

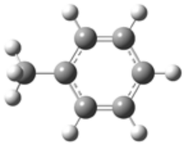

Center Number	Atomic Number	Atomic Type	Coordinates (Angstroms) X	Y	Z
1	6	0	-0.186699	1.245434	-0.008052
2	6	0	1.184666	1.242020	0.001668
3	6	0	1.888086	-0.000369	0.006755
4	6	0	1.184201	-1.242333	0.001637
5	6	0	-0.187139	-1.244993	-0.008058
6	6	0	-0.917199	0.000509	-0.016605
7	1	0	-0.743114	2.174892	-0.012595

8	1	0	1.743101	2.169362	0.005057
9	1	0	2.972098	-0.000565	0.016170
10	1	0	1.742165	-2.169951	0.005008
11	1	0	-0.744054	-2.174177	-0.012556
12	6	0	-2.398866	0.000253	-0.000377
13	1	0	-2.817838	-0.895971	-0.468574
14	1	0	-2.735812	-0.011109	1.057113
15	1	0	-2.818836	0.904396	-0.451432

Zero-point correction= 0.125866 (Hartree/Particle)

Thermal correction to Energy= 0.132377

Thermal correction to Enthalpy= 0.133321

Thermal correction to Gibbs Free Energy= 0.095092

Sum of electronic and zero-point Energies= -271.147682

Sum of electronic and thermal Energies= -271.141171

Sum of electronic and thermal Enthalpies= -271.140227

Sum of electronic and thermal Free Energies= -271.178456

N_3 radical

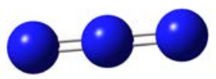

Center Atomic Atomic Coordinates (Angstroms)

Number	Number	Type	X	Y	Z
1	7	0	0.000000	0.000000	1.188344
2	7	0	0.000000	0.000000	0.000000
3	7	0	0.000000	0.000000	-1.188344

Zero-point correction= 0.009389 (Hartree/Particle)

Thermal correction to Energy= 0.012157

Thermal correction to Enthalpy= 0.013101

Thermal correction to Gibbs Free Energy= -0.012540

Sum of electronic and zero-point Energies= -164.135532

Sum of electronic and thermal Energies= -164.132764

Sum of electronic and thermal Enthalpies= -164.131819

Sum of electronic and thermal Free Energies= -164.157460

Benzyl cation

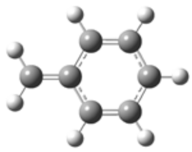

Center	Atomic	Atomic	Coordinates (Angstroms)		
Number	Number	Type	X	Y	Z
1	6	0	1.804058	0.000034	0.000209

2	6	0	1.122212	1.236059	0.000137
3	6	0	-0.254585	1.246633	-0.000294
4	6	0	-0.254516	-1.246664	-0.000162
5	6	0	1.122280	-1.236027	-0.000022
6	1	0	2.890903	0.000047	0.000224
7	1	0	1.684427	2.163464	0.000285
8	1	0	-0.807000	2.181651	-0.000715
9	1	0	-0.806901	-2.181699	-0.000241
10	1	0	1.684553	-2.163398	-0.000137
11	6	0	-2.355465	-0.000033	0.000131
12	1	0	-2.923053	0.927440	0.000724
13	1	0	-2.923391	-0.927306	0.000804
14	6	0	-0.983906	-0.000035	-0.000157

Zero-point correction= 0.117072 (Hartree/Particle)

Thermal correction to Energy= 0.122734

Thermal correction to Enthalpy= 0.123679

Thermal correction to Gibbs Free Energy= 0.087914

Sum of electronic and zero-point Energies= -270.560284

Sum of electronic and thermal Energies= -270.554621

Sum of electronic and thermal Enthalpies= -270.553677

Sum of electronic and thermal Free Energies= -270.589441

N_3H

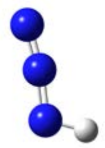

Center Number	Atomic Number	Atomic Type	Coordinates (Angstroms) X	Y	Z
1	7	0	0.142323	-1.124208	0.000000
2	7	0	0.000000	0.111236	0.000000
3	7	0	-0.304506	1.208105	0.000000
4	1	0	1.135282	-1.365935	0.000000

Zero-point correction= 0.021326 (Hartree/Particle)

Thermal correction to Energy= 0.024558

Thermal correction to Enthalpy= 0.025502

Thermal correction to Gibbs Free Energy= -0.001675

Sum of electronic and zero-point Energies= -164.772724

Sum of electronic and thermal Energies= -164.769492

Sum of electronic and thermal Enthalpies= -164.768548

Sum of electronic and thermal Free Energies= -164.795725

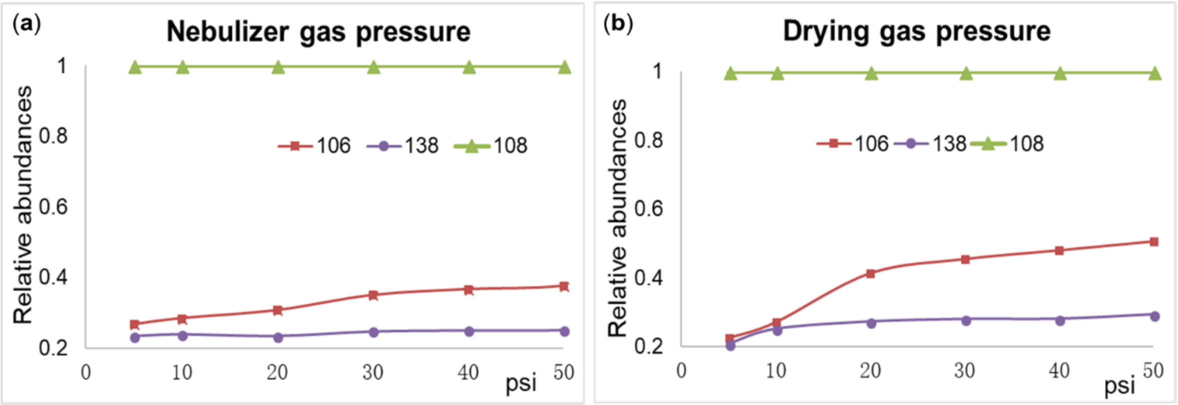

Figure S6. Relative abundances of the unusual product ions under different nebulizer gas pressure (**a**) and drying gas pressure (**b**)

Discussion: As shown in **Figure S6**, the relatvie abudances of *m/z* 106 and *m/z* 108 shows an obvious upward trend as the N_2 gas pressure increases, which intuitively indicates the key role of N_2 during the formation of these two unusual ions. This indicats that N_2 gas is likely to participate in subsequent gas-phase reactions during the ionization process

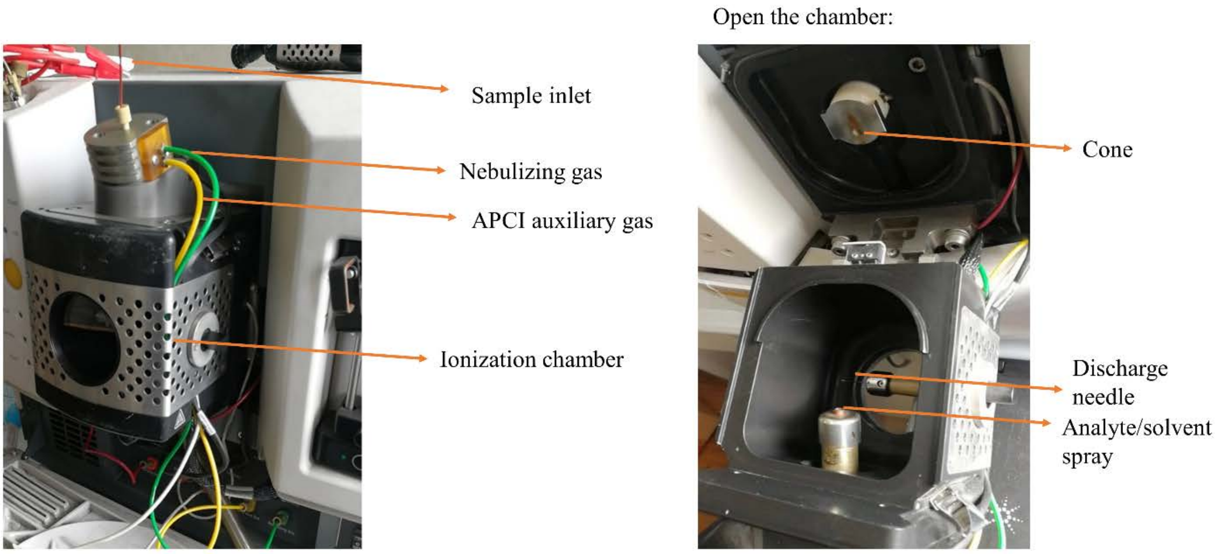

Figure S7. Photos of APCI-IT-MS and corona discharge apparatus
